# The harder the prep, the harder the recovery: a qualitative exploration of physique athlete perspectives on competition weight loss and restoration

**DOI:** 10.1080/15502783.2025.2576238

**Published:** 2025-10-17

**Authors:** Claire Buechel, Kate Pumpa, Naroa Etxebarria, Michelle Minehan

**Affiliations:** a University of Canberra, Research Institute for Sport and Exercise, Canberra, Australia; b University College Dublin, Institute for Sport and Health, Belfield, Ireland

**Keywords:** Bodybuilding, low energy availability, energy restoration, post-competition recovery, reverse dieting, recovery dieting

## Abstract

**Background:**

There is growing recognition of Low Energy Availability (LEA) symptoms in physique sports, however there are no clear recovery guidelines. This study explores how athletes perceive and manage recovery from prolonged and intentional LEA. Findings will inform future recovery strategies aimed at restoring energy availability.

**Methods:**

Semi-structured interviews were conducted with 19 natural physique athletes (11 males, 8 females) from Australia, New Zealand, North America, and Europe, and data were analyzed thematically.

**Results:**

Five themes were identified relating to weight management experiences pre- and post-competition: (1) pride, suffering, and rationalizing LEA, (2) navigating energy restoration, (3) body image disruption, (4) evolving autonomy, and (5) opportunities for supporting recovery. Perceived recovery was influenced by the severity of energy restriction, coaching support, and athlete readiness. Athletes voiced that psychological flexibility and physiological literacy were interconnected with successful outcomes.

**Conclusions:**

Athletes experience benefit from early recovery planning, applying flexible approaches to nutrition and training post-competition, and a shift from aesthetic to functional goals. Identified themes support treating recovery as a deliberate and individualized phase within the competitive cycle, with further investigation needed on optimizing post-LEA refeeding and coaching practices.

## 1. Introduction

Physique athletes engage in prolonged periods of low energy availability (LEA) to meet specific body composition targets [1]. LEA arises when dietary energy intake is insufficient to meet the combined demands of physiological function and training [2]. When sustained or severe, LEA can impair endocrine, metabolic, cardiovascular, gastrointestinal, and psychological functions, collectively described under the framework of Relative Energy Deficiency in Sport (REDs) [3]. While acute LEA may be a calculated and necessary aspect of weight manipulation in sport, chronic LEA can be detrimental to health and performance [3–5]. Physique athletes often experience chronic LEA as an inherent consequence of striving for extreme leanness more so than improved performance [6–8]. As such, symptoms typically associated with REDs, such as fatigue, menstrual cycle irregularities, and disordered eating behaviors, may not be avoided but rather accepted, rationalized, and strategically managed, as part of the competitive process [9,10]. Therefore, risk mitigation and appropriate recovery strategies become critical elements of competitive cycles.

Bodybuilders experience significant health disruptions pre- and post-competition including metabolic suppression, hormonal disturbances, hedonic responses to food, and psychological challenges [11,12]. Adaptations to prolonged LEA prime body fat accretion when dietary energy availability is restored, which can compromise future health, performance, and sport longevity [13,14]. Research tracking bodybuilders for 2 months post-competition demonstrates challenges with disordered eating behaviors, rapid weight gain, body dissatisfaction, and anxiety as they navigate recovery [1,15,16]. During this time, athletes follow various recovery strategies such as “reverse dieting” (slow, controlled calorie, and weight increase), “recovery dieting” (immediate return to estimated maintenance calories based on target off-season body weight), or ad libitum eating [11,17]. However, there is no clear consensus on best recovery practices for LEA.

Complicating this, the concept of “recovery” after weight loss for competition remains ambiguous. Recovery post-competition has been described in the context of restored body weight, physiological functions (e.g. menstruation, libido), eating behaviors, and training performance [7,16,18]. A small number of qualitative studies highlight the psychological and behavioral complexity of post-competition recovery, with bodybuilding athletes reporting identity disruption, loss of structure, and heightened emotional volatility as they transition out of rigid dieting phases [19,20]. These findings point to a need to better examine how athletes interpret and navigate recovery strategies after prolonged energy restriction.

This study aims to explore how physique athletes perceive and navigate recovery from intentional, prolonged LEA. Using a qualitative approach, we sought to generate useful insights that could support broader athlete populations to inform behavioral change and recovery strategies for restoring energy availability. This work may also guide hypothesis generation for future quantitative studies.

## 2. Methods

### 2.1. Research design

This exploratory, qualitative study consisting of one-on-one interviews was informed by a social constructivist epistemology, recognizing that knowledge is co-constructed through interaction [21]. A qualitative approach is well placed to explore the individual and social complexities of weight management in physique sports.

### 2.2. Participants

Natural physique athletes, defined as individuals who compete in sanctioned, drug‑tested competitions, and abstain from performance‑enhancing substances in accordance with their federation’s anti-doping policies, were recruited using purposive and snowball sampling. An electronic flyer was circulated via e-mail and social media to recruit athletes who were as follows: (i) age 18+ years, (ii) prior experience of structured phases of extreme weight loss and recovery periods similar to those required for bodybuilding competitions, and (iii) prior experience competing at a state or higher level (Table 1). Participants were based in Australia, New Zealand, North America, and Europe. Ethics was approved by the University of Canberra Human Research Ethics Committee (HREC-11972). A total of 19 participants (11 males, 8 females) were recruited.

**Table 1.** Participant characteristics (*n* = 19).

Characteristics	*n* (%)
**Gender**	
Male	11 (58%)
Female	8 (42%)
**Primary Sport**	
Bodybuilding	11 (58 %)
Powerlifting	8 (42 %)
**Highest Level of Competition**	
State	3 (16%)
National	9 (47%)
International	7 (37%)
**Training Experience**	
3–5 years	5 (26%)
6–10 years	9 (47%)
11+ years	5 (26%)

### 2.3. Procedure

Semi-structured interviews were conducted via Zoom (45–90 minutes), exploring experiences with energy restriction, recovery, and associated beliefs. The interview guide was pilot tested with two sports dietitians, one coach, and three athletes to assess clarity, relevance, and appropriateness of language [22]. Feedback informed the refinement of questions to better capture the nuances of lived experience, with sub-prompts used to promote deeper reflection. While the guide provided structure, interviews remained participant-led, allowing flexible discussion [23].

### 2.4. Data analysis

Audio recordings were transcribed using Zoom transcription software and checked for accuracy by CB. Reflexive thematic analysis was used to identify themes [24]. CB and MM independently read and coded transcripts before consolidating codes through discussion [25]. Themes were critically reviewed by all authors to ensure data reflected the voices of participants [26]. Data collection ceased when it was agreed that the sample had sufficient information power according to the elements outlined by Malterud et al. [27].

### 2.5. Trustworthiness

All authors have professional backgrounds in sports dietetics and/or exercise science. CB brings additional insider perspective through coaching and personal competition experience in strength and physique sports. This experience facilitated trust-building and information sharing [26]. To strengthen trustworthiness, we considered Braun and Clark’s guidance on quality reflexive thematic analysis [24]. This method supported exploration of content and underlying meaning, while recognizing the influence of the research team. Our research is reported in line with COREQ guidelines [28], though we recognize that some components (e.g. approaches to saturation and data coding) of COREQ do not fully align with the philosophical underpinnings of reflexive thematic analysis.

## 3. Results

Five key themes captured the experiences of physique athletes who undergo prolonged energy restriction for competition and navigate post-competition recovery.

### 3.1. Pride and suffering: rationalising the risks of LEA

Athletes viewed hunger, fatigue, and social sacrifice as a marker of dedication and success, “*You have chosen to do something extreme with an extreme adventure. You can expect extreme challenges*” (F3). This cultural framing of suffering obscured early signs of REDs, normalizing disordered behaviors and physiological disruptions, including loss of sleep, strength, reproductive function, and cognition function. Negative outcomes were framed as personal weakness or lack of commitment rather than signals of physiological harm, “*I couldn’t tell him that I was struggling because I didn’t want him to pull me out of the program and say, Well, you don’t have what it takes.”*(M4). Athletes accepted symptoms of LEA, viewing them as calculated trade-offs for competitive success: “*When I decide to compete in bodybuilding, I am making an intentional departure from certain aspects of my health”* (M5). Similarly, F4 framed the acceptance of harm as bounded by time, “*I can accept being unhealthy because it’s such a short period of time. For me, it was always, ‘How far can I push this?’ And then, the moment I’ve done it, let’s get out and get back to being healthy*.” Some athletes acknowledged the recklessness of their early behaviors, “*I threw myself so brutally at training, at everything, and I didn’t really care about my mental health”*(M2).

This willingness to endure harm was reinforced by a reliance on external coaching, particularly early in athlete’s careers. Athletes described coaching relationships and peer environments that reinforced rigid discipline and sometimes discouraged questioning. As F4 explained, “*I just do whatever coach tells me, to the point where I don’t even want to question it. I don’t ask him, because I don’t want to put doubts on him, I just need him to look after it. It’s just one less thing to worry about*.” While this reliance could reduce decision-fatigue, it also led some to persist with harmful strategies despite clear signs of dysfunction, “*Coach put me on Keto, where it was like a hundred percent protein. No carbs. Carbs are the enemy … that’s when I looked emaciated, I was barely functioning*” (M4). Athletes hesitated to question their coaches for fear of appearing weak or non-compliant.

The ability to endure and control suffering was central to how athletes defined success and self-worth. They expressed pride in aspects of energy restriction where they felt every decision was purposeful: *“It’s perfectly tailored and orchestrated, from the second you wake up to the moment you go to bed. Every single choice is making you better. Not just from an aesthetic standpoint; it’s making your digestion better, your sleep better … ”* (M2). While challenging, dieting was embraced as an opportunity to test resilience, “*I have the physical resilience, but I wanted to prove that I have the mental resilience*” (M11). This sense of purpose evolved into a relentless commitment that persisted even as restriction became extreme. M2 described how his dedication continued to drive him as the process shifted from efficiency to managing suffering, “It tips over just a bit too far, and everything gets f***ing awful. But you do it. I would have gotten my cardio in before my mum’s funeral, there was nothing that was going to stand in the way.” For these athletes, the mental battle reinforced value of suffering in the pursuit of competitive success.

### 3.2. Navigating energy restoration and recovery

The immediate aftermath of competition was described as just as challenging as the weight loss phase itself. The transition from disciplined to normal eating was complex, involving rapid weight gain, intense food focus, digestive issues, and psychological challenges. Athletes described a direct relationship between the severity of their weight loss phase and the difficulty of their recovery. The more rigid and extreme their weight loss strategies, the more challenges they often faced after competition, *“The amount that I rebound decreases each year, and that seems correlated with how I can minimize the stress required to get into stage shape. It’s like the amount of restriction that I feel is inverse to the amount of rebound that I have”* (M5).

Recovery was further challenged by a combination of metabolic dysfunction and psychological disorientation following prolonged restriction. Participants described losing structure and direction once the rigid contest prep phase ended: “*After having such rigidity around variables, it can be hard to find purpose in maintaining that rigidity. When arguably, it’s a really important time … the most challenging thing can just be having purpose around nutrition”* (M9). This loss of purpose alongside physiological drivers to restore weight was identified as contributors to rebound eating, “*Your body’s naturally going to be geared towards gaining weight. You can overshoot it. You need to set some degree of loose structure for good habits moving forward*” (F4).

Participants described experimenting with various recovery strategies; however, no single approach was considered universally effective. Successful recovery appeared more dependent on psychological readiness, clarity of post-competition goals, and prior experience more than any specific nutrition strategy. Even when nutrition strategies such as reverse or recovery dieting were implemented to control the rate of weight regain, athletes struggled with an overwhelming drive to eat and episodes of binge eating. As F3 noted, *“You might think you’re ready, but there is something that overcomes you, you think you’re just going to have one cookie. You think you can be smart and not have a second. It does not work like that”*. With the sudden decrease in dietary rigidity, self-control became exceedingly difficult:

I blew up immediately, I’d have massive bloat going on, I’d be walking around as if I’m pregnant, and I got my hands on my lower back just breathing heavily. But even in those moments, your body still signals you to eat more. And it’s like, you feel like crap, you know what’s going to make you feel better, more ice cream (M4).

In some cases, athletes reported purging, not from guilt, but to relieve extreme abdominal pain caused by overeating, “I was like, I’ve f***ed up, I’m in agony, and I want some sweet relief”(M2).

Disrupted hunger cues and food obsession often persisted months after competition, raising questions about the timeline for physiological recovery. For some, normalized food focus and indicators of hormonal regulation (libido, menstruation) signaled the turning point toward recovery. For example, F4 reported her disordered levels of food focus did not relent until her menstrual cycle returned: “P*recisely 3 months to the day of my last Comp something happened, and I felt completely different. My period came that day. My hormones just felt normal, and I felt like I was back in control.”* However, many lacked clarity on what marked full recovery. Participants noted that time was a key factor in their recovery. While structured nutrition provided a sense of control, full recovery was described as a multifactorial process, driven by gradual physiological and psychological adaptation in the context of sustained energy restoration. This insight reinforced that recovery was not linear or uniform.

### 3.3. Body image disruption after weight loss

Despite understanding the need for weight restoration, athletes described significant body dissatisfaction, guilt, and anxiety post-competition, “*Just going from where you look in the mirror … your weight dropping every day … you’re skeletal, too thin, but it’s normal … all of a sudden you put on 1 kilo, and it feels like you put on 10”* (F7). These concerns were tied to identity and social expectations to stay lean: “*There was definitely pressure to maintain extremely low body fat percentage … it was expected … you’re a bodybuilder; you just maintain that incredible looking physique year-round”* (M4). Similarly, it was common for external messaging to amplify internal doubt: *“When people say, oh, are you training now? And I’m like, well I’ve always been training. I’m just not dieting now for a competition … it gets to you”* (F7). This was also reflected by M6, *“People will say, oh, you’re not as lean anymore. It messes with your head, even when you logically know that this is part of the process”*.

Fear of excessive weight gain was closely tied to disordered eating behaviors, as F1 described how purging became a method of attempted weight control:

I was extremely scared of seeing my body fat increase … I would just taste a food, and then one bite would turn into 2, 3, 4, and then I’d be like f***, I’m just going to eat the whole thing and then go make myself sick. So, that’s when the purging came into play … and when I realized I had a problem.

Some athletes coped with body composition changes through reframing negative self-talk. F1 described changing her language, catching moments of harsh self-judgment, “*where I’d be like, God! Look at your legs! They’re so fat! Or look at your stomach that’s hideous …* ,” and shifting to focusing on gratitude, “*you’ve housed two little humans that are perfectly healthy*.” Athletes also worked to reassess what “healthy” or “ideal” body composition looked like, *“After three consecutive months of not getting stronger from trying to stay lean, I realized I had to start eating more. I changed my opinion on the ideal male physique. I want to look like a thick meat truck”* (M1), and F7 echoed, *“I began to enjoy the way I looked as I was putting weight on. I was looking bigger and fuller, training’s good again, you feel strong. Each year it gets easier to embrace the fluffy stage”*. Additionally, some navigated this discomfort with weight regain by moving up a weight class or into a different competition classification. Adopting a new weight goal linked to future competition helped rationalize weight gain.

### 3.4. Evolving autonomy and the role of macro tracking

As athletes progressed in their careers, they described a transition from passive adherence toward more reflective and autonomous approaches to weight management. They explained the shift was driven by negative experiences involving burnout, underperformance, disordered behaviors, or poor coaching guidance. Athletes spoke about developing autonomy through critical reflection on early mistakes and trial-and-error:

When I started, I was like, it doesn’t matter how I feel. I was your typical boneheaded, all-in athlete. I realized that good athletes have a high degree of body awareness and self-reflection. Not a I don’t care about any of that; I’ll drink blood to get in shape … I realized that a “bull in the china shop” mentality wasn’t the way you got to higher level performance (M5).

Most athletes described this learning process as necessary and inevitable, “*If I ate different, I looked different. If you think critically and are curious, you start to question people you thought should be in the know. Your body will always tell the truth, regardless if you want to listen*” (F3). For others, mistakes were framed as essential stepping stones to progress: *“I bombed completely. But I learned from it. That’s what you have to do in these sports. There are no shortcuts. It’s just continuous learning all the time … Make the mistakes early, you’ll get better over time*” (F5).

Athletes typically began their journeys with strict macronutrient tracking, which served as both a valuable entry point into understanding energy balance and a potential source of mental strain. M7 identified it as a foundational skill:

Tracking is a good utility to have. Using it when we’re dieting can be pretty good, and even after dieting to not get like a rebound effect. It’s nuanced, but I think it’s a skill that everyone should have. It can foster a lot of flexibility, which is kind of where a lot of dieting goes wrong because they will impose rules and restrictions.

However, some found long-term reliance on tracking psychologically exhausting. M9 shared, *“I had a lot of phases where I got tired of tracking my nutrition, and tired of being so intense with it. Sometimes I would ask for a month off from tracking, I think it depends on your mindset”*. M5 echoed this, describing his shift toward more sustainable nutrition practices, *“the fact that I have to manage the stress of tracking means it’s a stressor. If I can unload some stressors and be just as good, if not better, then that is very facilitative for me.”*

Athletes emphasized the benefits of making autonomous decisions; however, acknowledged this brought on different stressors. *“You have to navigate fact from fiction and apply it to your own context. Look at nutrition; how many different ways of eating are there? People get obsessive; one way of eating is the only way, it’s religious”* (F3). Athletes voiced that greater autonomy fostered individualization and flexibility, however it did not always improve physiological literacy or recovery outcomes. They described remaining vulnerable to symptoms associated with prolonged LEA, even when their approaches felt more intuitively appropriate.

### 3.5. Opportunities for supporting recovery

Athletes described a range of strategies to navigate the post-competition period and support recovery from prolonged LEA. No universal optimal approach was identified. However, post-competition strategies centered around managing the rate of weight regain, mitigating digestive distress, improving performance, and supporting psychological acceptance of body composition changes.

Athletes who experimented with reverse dieting noted how high cognitive demands and the slow timeline of this method made it difficult to sustain: “I tried reverse dieting, but I couldn’t sustain it. I was just so f***ed up with everything that I went straight back to maintenance calories. I’m just going to eat up to the point that I feel comfortable with” (F8). Others immediately returned to their pre-restriction intake (recovery diet), although still experienced heighted food focus and hunger: “*We did the recovery diet as opposed to the reverse. But that doesn’t stop you from feeling like you’re starving for a little while”* (F4). Even when energy intake increased, time rather than dietary precision appeared most important:

Your stomach needs time to go back to what it thinks is normal. You’ve taken your body to an extreme. It doesn’t matter how much food you throw at it. The whole system needs time to adapt back. (F3)

Athletes emphasized that coaching support should extend into the post-competition period. While coaches were often heavily involved during competition prep, they were sometimes absent or less engaged post-competition when athletes needed the most guidance and reassurance. Some described positive post-competition experiences where their coach helped facilitate intuitive eating and self-trust: *“When I would check in it would be: Are you hungry? Yes, eat more. The following week: Are you hungry? Yes, eat more. So I would, and just through listening to my body, my performance started increasing”* (F1). Athletes who later transitioned into coaching roles themselves reflected on the importance of supporting long-term health and sustainable progress: “*We’ve got a duty of care to make sure that people aren’t being reckless with their behaviours”* (M9).

Athletes described moving from aesthetic goals to performance-based goals to help reframe weight gain as functional rather than undesirable. A shift between bodybuilding and powerlifting was common between bodybuilding competition periods, *“I shifted to just being stronger and developing the skill of lifting. Powerlifting was a big thing for me because it took the focus away from being judged on how you looked to what you can achieve”* (F1). Reportedly, this approach also helped manage emotional challenges and prevent disengagement. M1 described how redefining his motivations post-competition helped him regulate his behaviors, “*I think it’s important that your motivation is strong enough to get you through those post-competition barriers. My motivation to walk on the beach and feel comfortable was higher than any desire for food*.”

Most athletes emphasized that recovery was successful when it felt intentional and guided by internal motivation rather than external pressure. As M5 reflected, “*I didn’t want to feel like this process was happening to me, but rather that I was actively engaging in it. I want to gain weight quickly, but not like super-fast.”*

## 4. Discussion

This study documents how physique athletes experience and manage recovery following prolonged LEA. Perceived recovery was shaped by participants’ reactions to biological responses (e.g. hyperphagia, hormonal disruption, and weight gain), identity challenges, prior experience, and the quality of coaching support. Flexible dietary approaches, autonomy-supportive coaching, and recovery-specific education were viewed as positive, underscoring the potential importance to treat recovery as a structured, coach-supported phase addressing both physical and psychological factors.

Physique athletes experienced varying physiological symptoms during recovery including hyperphagia, gastrointestinal distress, rapid weight gain, disrupted sleep, and disordered eating behaviors, regardless of increased caloric intake. Such experiences align with established models of adaptive thermogenesis where metabolic adaptations, induced during chronic LEA, continue beyond the cessation of dietary restriction [29]. These adaptations are considered to promote efficient energy conservation and fat restoration upon refeeding through suppressed leptin and thyroid hormones, elevated ghrelin, and increased dopaminergic response to food, all of which promote energy storage and hedonic eating behaviors [30,31]. Additionally, reductions in adipocyte size during caloric restriction without a loss in cell number contribute to efficient lipid re-accumulation during refeeding [32]. Consequently, athletes may interpret these biologically driven symptoms of rapid weight gain or heightened food drive as personal failures rather than expected compensatory responses, driving psychological distress during recovery.

To mitigate rapid weight regain post-competition, participants often implemented reverse (incremental caloric increases, e.g. 100kcal per week), or recovery dieting (a more immediate return toward estimated target body weight maintenance calories). While reverse dieting is popular within physique sport communities to control rate of fat regain, empirical evidence remains scarce. A recent scoping review found no consensus on optimal refeeding strategies for physique athletes post-competition, underscoring the need for more comparative trials in this area [11]. In practice, many participants found reverse dieting unsustainable due to its high cognitive demand, rigidity, and the burden of ongoing self-monitoring post-competition. These findings align with literature demonstrating that rigid dietary control is associated with heightened food preoccupation, disordered eating behaviors, and greater body image concerns [33]. Conlin et al. [34] found that resistance-trained athletes following flexible dieting approaches had greater fat-free mass gains over 10 weeks of ad libitum eating post-diet compared to the rigid dieting group (1.7 kg vs. 0.7 kg, respectively). These findings suggest that while reverse dieting may offer athletes a sense of structure and control, ongoing restraint and self-monitoring may unnecessarily prolong LEA and psychological distress, without providing benefits to post-diet body composition.

In contrast, recovery dieting that follows more flexible frameworks, such as portion-based eating, macronutrient ranges, or autoregulated intake, was generally perceived by participants as more physiologically and psychologically supportive. This aligns with REDs guidelines recommending prompt energy restoration [3], and with recommendations for physique athletes to regain weight at a rate that facilitates full recovery, while avoiding deposition of excessive fat mass in the early phase of weight regain [35]. The increased caloric flexibility afforded by recovery dieting fosters dietary flux, adaptive eating behaviors, and improved emotional regulation [36,37]. By encouraging responsiveness to internal cues such as hunger, fatigue, and satiety, recovery dieting may better support sustainable recovery outcomes in comparison to approaches requiring sustained surveillance. Additionally, recovery dieting may facilitate both energy restoration and meaningful increases in fat mass. In a case-series of natural physique athletes, Longstrom et al. [14] reported positive correlations between RMR, leptin, and fat mass regain during 10 weeks of post-competition refeeding. Athletes employed a range of post-diet strategies, resulting in variable degrees of fat gain, highlighting the physiological importance of adequate weight restoration. According to Speakman’s dual intervention point model, recovery requires surpassing a lower threshold of body fat to restore leptin signaling and metabolic functioning, mechanisms that are more strongly linked to fat mass than energy availability [38,39]. This may explain why participants perceived recovery dieting as more supportive than reverse dieting strategies aimed at minimizing fat gain.

Despite understanding the physiological need for post-competition weight restoration, athletes experienced significant distress related to post-competition body composition changes. While this likely stems from the internalization of aesthetic ideals where peak leanness is central to identity and perceived self-worth, athletes were also concerned over performance-based consequences of excessive fat gain. Increased body fat was frequently accompanied with shame, self-criticism, and perceived social scrutiny, reflecting aesthetic identity salience where self-evaluation is anchored in physical appearance [40,41]. However, in a sport where achieving and maintaining low body fat is directly tied to competitive success, some distress may be a rational response to overshooting a perceived ideal position for offseason adiposity. These dual sources of distress, identity-driven and performance-driven, highlight the need for nuanced support strategies that address both psychological and performance-related concerns. For example, athletes who reframed post-competition weight gain as instrumental for strength, muscle growth, or recovery, reported greater psychological resilience and improved body acceptance. This realignment of functionality over aesthetics aligns with Ricketts et al. [42] who found that functionality appreciation enhances self-efficacy and well-being in athletic populations. Furthermore, self-compassion and positive self-talk were used by participants to buffer body-related distress during this transition. Such strategies have identified by Killham, Kowalski, and Duckham [43] showing self-compassion cultivated more realistic performance standards and improved body image in female athletes. Transitioning away from a competition-centered identity is not always intuitive or linear, particularly for those whose self-worth is closely tied to physique-based achievement. Therefore, supporting the development of more flexible, performance-centered identities that prioritize function, health, and long-term well-being is a key objective for recovery.

Athletes with prior recovery experience described improved psychological outcomes and greater behavioral flexibility during the recovery process. Symptoms such as extreme hunger, food focus, or isolated binge episodes were reframed as predictable consequences of prolonged LEA rather than personal failures to adhere. This perspective is supported by Langbein et al.’s findings that psychoeducation and anticipatory coping strategies help athletes manage distress during recovery by preparing for the cognitive and physiological shifts involved [44]. Athletes who lack understanding of internal cues, such as hunger, fluid, or hormonal shifts, may interpret these signals as signs of failure, triggering increased resistance to weight gain and compensatory restriction or rigidity. These patterns demonstrate that effective autonomy must be scaffolded to support sustainable motivation and behavior throughout recovery [45]. Athletes also described learning through trial and error, with repeated exposure to the recovery process helping to develop body trust, interoceptive awareness, and cognitive flexibility, traits shown to buffer against disordered eating and psychological distress [46,47]. Structured psychoeducation during the weight loss phase may help athletes contextualize future recovery symptoms, reduce shame, and foster the competence required for authentic autonomy.

Coaching support was viewed as important for psychological safety during recovery. Among novice athletes, this support often translated to high levels of dependency, with individuals deferring to prescriptive coaching despite emerging signs of physical or psychological harm. This pattern reflects findings on hierarchical coach – athlete relationships, where authoritarian coaching dynamics can suppress reflection, discourage help-seeking, and normalize rigid discipline, perpetuating disordered behaviors [48–50]. Complicating this, high-performance sport culture often prizes stoicism leading athletes to conceal distress to preserve a facade of resilience [51,52]. Within this context, suffering can be equated with commitment and success, contributing to the delay recognition of dysfunction and accessing timely support. Participants also observed a notable decline in coaching engagement following competitions, despite this period being marked by heightened physiological and psychological vulnerability. Without continued guidance, athletes struggled to interpret markers of recovery and expected biological responses to chronic LEA such as changes in reproductive function, appetite regulation, and body weight. This is a challenge for both athletes and coaches as LEA recovery is highly individual and non-linear, with no clear biomarkers or expected timelines to guide the process [3,53]. Given the highly individualized nature of LEA recovery, continued coaching beyond competition is essential to help athletes navigate the physiological and psychological transitions of the recovery process.

Participants who received ongoing, autonomy-supportive coaching described more adaptive and successful recovery experiences. These coaching styles normalized post-competition changes, encouraged intuitive eating, prioritized health, and supported shifts toward performance-based goals. These findings align with Helms et al., who emphasize the need for post-competition coaching styles that incorporate gradual nutritional transitions, psychoeducation, and mental health-informed strategies [54]. Improved coaching standards regarding post-LEA recovery, including ongoing support, guiding physiological literacy, and incorporating harm-reduction strategies may improve long-term health and performance outcomes.

Participants in this study identified several practical strategies to support recovery from prolonged LEA. These centered around coaching continuity beyond competition, promoting flexibility, reframing recovery goals, and improving psychological readiness. Practical athlete-informed strategies are summarized in Table 2.

**Table 2.** Recommendations for athletes recovering from a period of prolonged, intentional LEA.

Category	Practical Recommendations
Proactive Recovery Education	Start recovery planning during the weight loss phase Guide athlete expectations for recovery (weight gain, food focus, identity shifts) Build self-awareness & adaptability Involve athletes in planning & decision-making Co-create harm reduction & exit strategies
Goal & Identity Reframing	Have early conversations about post-competition goals Frame recovery as an equally valuable goal-oriented phase Support identity growth beyond physique Challenge the idea that success requires rigidity; flexibility can also foster success
Post-Competition Coaching	Maintain coaching involvement post-competition Provide a clear recovery plan Offer emotional support Monitor ongoing LEA symptoms Help athletes understand signs of recovery (e.g. appetite regulation and hormonal function)
Flexible Approaches	Incorporate flexible nutrition strategies, e.g. macro ranges and auto-regulated refeeding Guide athletes to adjust food and training based on day-to-day needs and stress levels

### 4.1. Limitations

This study offers qualitative insights shaped by the cultural context of participants and researcher interpretation. Participants were intentionally selected based on having multiple experiences with energy restriction and recovery, allowing deeper exploration of how perceptions and strategies evolved over time. While this approach provided valuable insights, it may have excluded less experienced athletes. Although the sample included participants from diverse geographic regions, intersecting factors such as gender, age, and cultural background were not central to analysis and warrant further exploration in how they shape experiences. In particular, recovery strategies may differ by sex, especially in relation to hormonal regulation (e.g. menstrual function) and disordered eating risk. While REDs affect both male and female athletes, women face greater risks for reproductive suppression and bone loss, necessitating sex-specific monitoring and tailored interventions [3]. Although findings provide insight into athlete experiences and preferences regarding recovery strategies, the efficacy of reverse or recovery dieting approaches remains untested.

## 5. Conclusions

Athlete recovery from intentional, prolonged LEA for competition is a complex, multifaceted process involving physiological restoration, psychological adjustment, and identity renegotiation. This study highlights how recovery outcomes are shaped by biological responses to chronic energy restriction, individual physiological literacy, psychological flexibility, and levels of coaching support. Recovery was perceived as more successful when athletes were supported by autonomy-supportive coaches, proactive education, and environments that valued flexibility, self-compassion, and long-term well-being. These findings underscore the importance of considering recovery as a critical, coach-supported phase that is intentionally planned and individually tailored. Future research should explore personalized recovery models and test the efficacy of specific refeeding strategies to optimize both health and performance outcomes.
